# Tuning gut microbiota through a probiotic blend in gemcitabine‐treated pancreatic cancer xenografted mice

**DOI:** 10.1002/ctm2.580

**Published:** 2021-11-04

**Authors:** Concetta Panebianco, Federica Pisati, Maria Ulaszewska, Annapaola Andolfo, Annacandida Villani, Federica Federici, Manna Laura, Eleonora Rizzi, Adele Potenza, Tiziana Pia Latiano, Francesco Perri, Claudio Tripodo, Valerio Pazienza

**Affiliations:** ^1^ Gastoenterology Unit Fondazione IRCCS “Casa Sollievo della Sofferenza Hospital, San Giovanni Rotondo Foggia Italy; ^2^ Histopathology Unit Cogentech S.C.a.R.L Milan Italy; ^3^ Proteomics and Metabolomics Facility (ProMeFa) IRCCS San Raffaele Scientific Institute Milan Italy; ^4^ Sintal Dietetics s.r.l. Castelnuovo Vomano Teramo Italy; ^5^ Dietetic and Clinical Nutrition Unit Fondazione IRCCS Casa Sollievo della Sofferenza San Giovanni Rotondo; ^6^ Oncology Unit Fondazione IRCCS “Casa Sollievo della Sofferenza Hospital, San Giovanni Rotondo Foggia Italy; ^7^ Tumor Immunology Unit Department of Health Sciences University of Palermo Palermo Italy

Dear Editor:

An increased belief of the therapeutic effect of probiotics has recently risen, since their consumption, by restoring a healthy gut environment, prevents cancer onset and progression and also boosts anticancer therapies’ efficacy while reducing their toxicity.^1^ We previously uncovered that gemcitabine deeply modifies the intestinal microbiota in a mouse model of pancreatic cancer (PC), shifting it toward an inflammation‐related profile.^2^ Based on this observation, we formulated a probiotic blend (composed as in Table S1), which was tested in the current study to investigate whether restoring a balanced microbiota could reduce gemcitabine‐induced side effects.

The effects of gemcitabine (GEM), probiotics (PRO2101) and gemcitabine+probiotics (GEM+PRO2101) treatments on PC mice were investigated compared to nontreated mice (CTRL), according to the protocol in Figure 1A. Treatments tended to decrease tumors’ growth without reaching statistical significance (Figure 1B). Animal body weight was monitored all along treatment, without recording significant changes among groups (data not shown). Figure 1C shows histological examination of tumor tissues. A high‐grade malignancy with high degree of pleomorphism consisting of prismatic cells intermingling with spindle‐shaped cells was observed in CTRL, together with a high frequency of apoptotic figures. Ki‐67 immunohistochemistry revealed a proliferative fraction of 31.5%. Phospho‐H2A.X staining, indicating DNA damage and late apoptosis, was absent (0%). On Picrosirius and Masson's trichrome stromal bundles were characterized by dense collagenic deposition. Upon GEM treatment, the cell morphology appeared less pleomorphous with a predominance of spindle‐shaped elements, but no significant reduction in the proliferative index was observed (29.7%). Phospho‐H2A.X staining was more abundant (41.8%) and the collagen fibers displayed a looser arrangement compared to control. In PRO2101 samples tissue morphology was similar to CTRL, with a relative enrichment in prismatic cells. Frequent apoptotic figures, an intense phospho‐H2A.X staining (76.2%) and a slight reduction of proliferating cells (15.9%) were observed, together with an overall reduction in stromatogenesis. In GEM+PRO2101 mice group, the morphology of malignant cells was less pleomorphous with a predominance of spindle‐shaped elements. Ki‐67 staining (30.3%) and the stromal reaction were comparable to those of CTRL. DNA damage was markedly increased (94.5%) with respect to either GEM and PRO2101.

**FIGURE 1:**
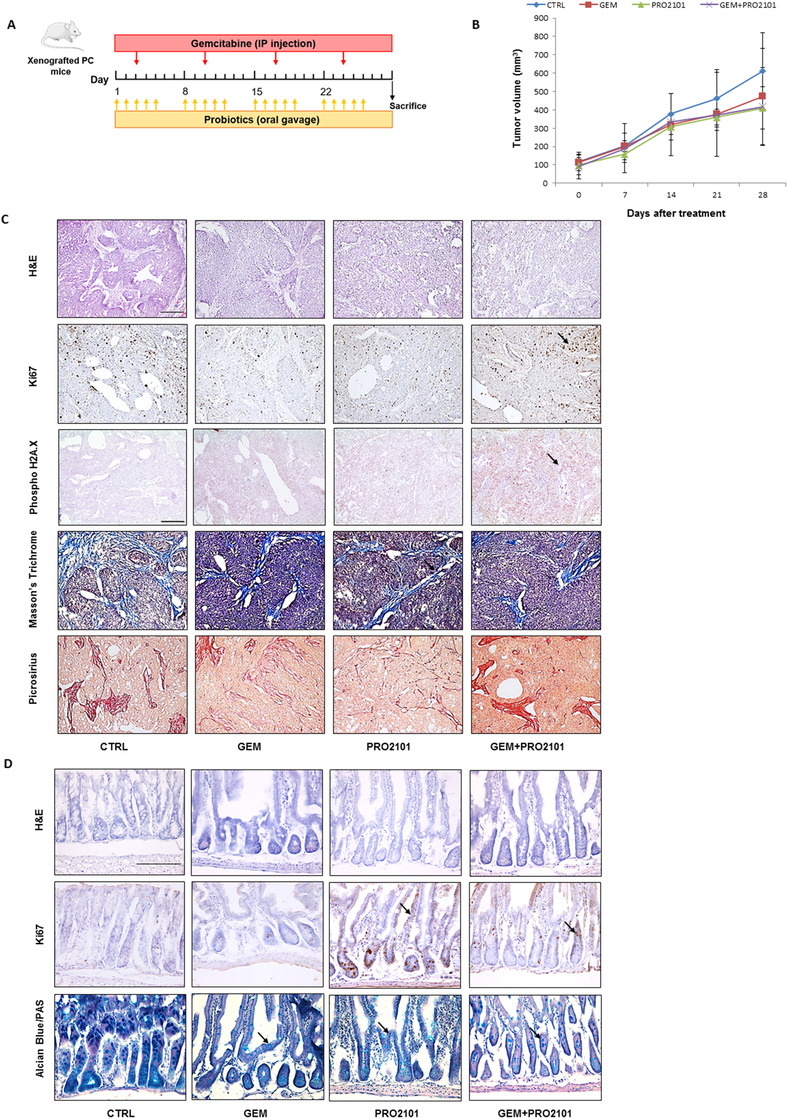
Impact of gemcitabine and/or probiotic treatment on pancreatic cancer growth and histology and on intestinal mucosa structure. (A) Schematic representation of the experimental protocol for treatment of pancreatic cancer mice with gemcitabine and probiotics. (B) Tumor volume growth curves of mice subjected to different treatments or not treated (*N* = 5). Data are shown as mean ± SD. (C) Histological stain of tumors with H&E, Ki67, Phospho‐H2A.X, Masson's Trichrome and Picrosirius Red. All pictures were taken at 10X magnification. Scale bar is 150 μm. (D) Histological stain of mouse intestinal sections with H&E, Ki67 and Alcian Blue/PAS. Pictures were taken at 20× magnification. Scale bar is 150 μm

Epithelial‐mesenchymal transition (EMT), assessed either by immunohistochemistry (Figure S1A) and by immunoblot (Figure S1B–C), revealed a milder mesenchymal phenotype upon probiotics administration. Details are reported in Supplementary results. We then wondered whether probiotics could have any protective effect on chemotherapy‐related intestinal damage (Figure 1D). Gut mucosa in CTRL was characterized by regularly spaced crypts with preserved mucus synthesis and a normally populated lamina propria. The few proliferating elements were normally localized within the crypts, coherently with a homeostatic condition. GEM samples showed a global reduction in mucin synthesis by epithelial cells lining the crypts, which had irregular nuclear morphology and altered chromasia. The surrounding stroma of the lamina propria showed signs of edema and a slight/focal increase in the inflammatory infiltrate. An overall decrease in proliferating elements suggested no epithelial regeneration. PRO2101 samples showed a decrease in mucin synthesis as compared with GEM. The lamina propria had signs of focal edema and increase in inflammatory cell density. The proliferative activity of epithelial cells was increased compared with both CTRL and GEM, which, along with the decrease in mucus secretion is suggestive of regenerative changes. In GEM+PRO2101 the overall architecture was better preserved as compared with GEM. Epithelial cells had a more regular morphology and partially preserved mucin content. An increase in inflammatory cell infiltration in the lamina propria was still observed. Ki‐67 was higher than that of a quiescent mucosa and the localization of proliferating cells was consistent with their regenerative attempt.

Microbiota analysis revealed that both PRO2101 and GEM+PRO2101 treatment increased the species richness, expressed by the Observed (Figure 2A) and the Chao1 index (Figure 2B), compared to the GEM group, in which it tended to drop with respect to CTRL. As for alpha‐diversity metrics, no variation was observed for Shannon (Figure 2C) and Simpson (Figure 2D) indices whereas the Fisher index significantly dropped in GEM versus CTRL and, it was restored by both PRO2101 and GEM+PRO2101 treatments (Figure 2E). The bacterial composition at the phylum, family, genus and species level in the four groups is represented in Figure 2F–I and described in Supplementary results. Bacteria producing butyrate and other beneficial short chain fatty acids, such as *Eubacteriaceae*, *Ruthenibacterium*, *Faecalicatena*, *Pseudobutyrivibrio*, and *Roseburia*, were enriched in mice receiving probiotics alone or in combination with chemotherapy.

**FIGURE 2:**
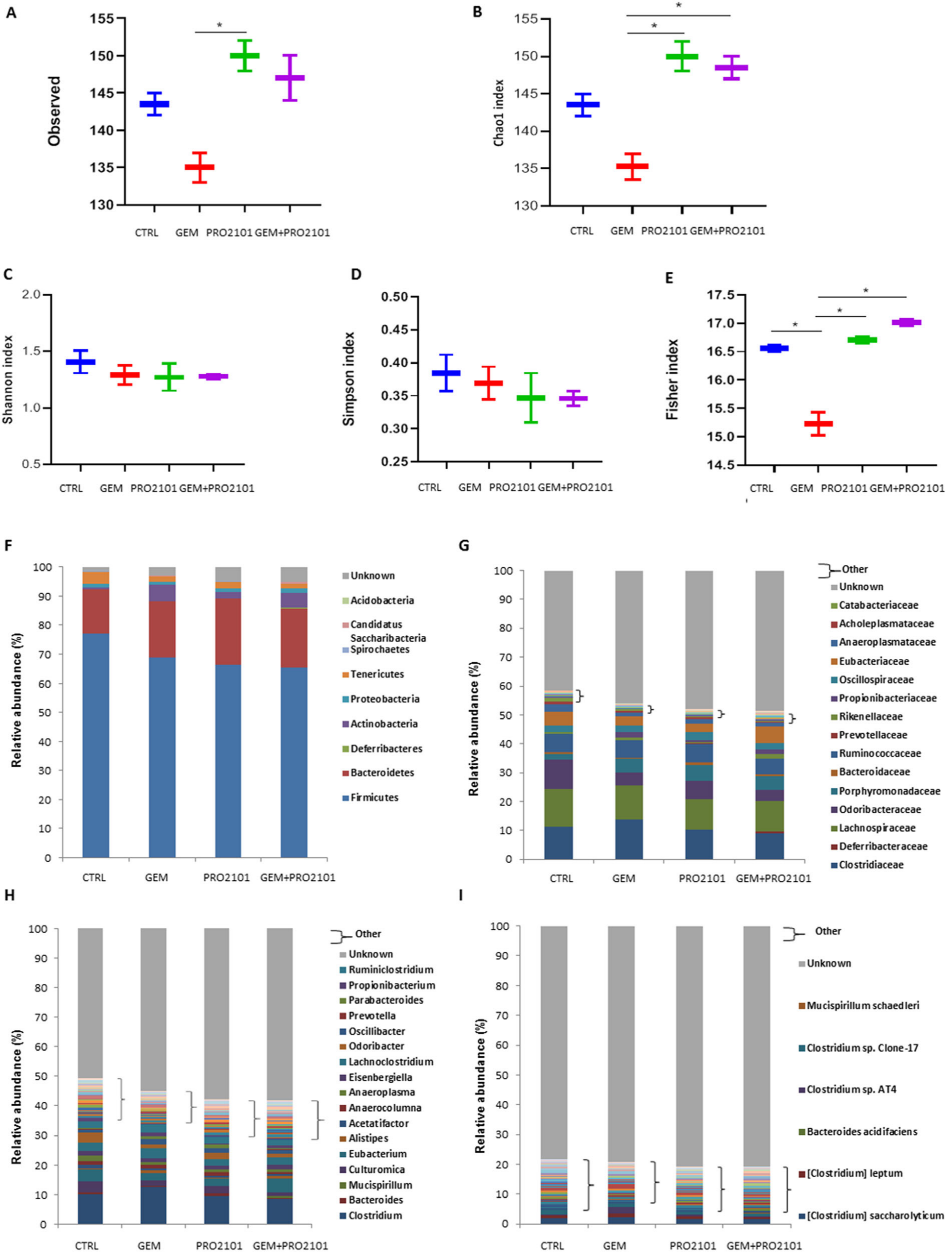
Gemcitabine and/or probiotic treatment shape gut microbiota richness and composition. Species‐level microbial richness as expressed by the Observed (A) and Chao1 index (B) and alpha‐diversity as expressed by Shannon (C), Simpson (D), and Fisher (E) indices. Mean relative abundance of gut bacteria at the phylum (F), family (G), genus (H), and species (I) level. Data are expressed as means of two fecal pools per experimental group. Differences were considered significant when *p* <  .05 (*)

We next evaluated the effect of probiotics on hematological, hepatic, and renal toxicity. Mean values for blood count and serum biochemical markers in each group are reported in Table S2 and detailed in Supplementary results. Briefly, GEM caused red cell count to drop below the normal range, which was abolished in mice cotreated with PRO2101. Furthermore, probiotics restored the platelet number, affected by gemcitabine.

Since the microbial–host cometabolism influences host physiological functions,^3^ metabolomic profiling of serum samples was performed. As shown from the PCA on annotated compounds (Figure 3A) CTRL and GEM samples tightly cluster in the upper left part of the plot, while PRO21010 and GEM+PRO2101 samples are more spread in the remaining part, indicating that probiotics introduce a higher variation to the serum profile. Figure 3B and 3C, respectively, report the heatmap representation of serum metabolites in 1 and 2a annotation levels, confirming the above‐described clusterization of probiotics‐receiving groups. A significant decrease in many amino acids, including alanine, tryptophan, tyrosine, lysine, leucine, and glutamic acid was observed in mice receiving probiotics compared to CTRL or GEM‐treated animals. Besides being fundamental for protein synthesis, amino acids provide atoms for lipid and nucleotide biosynthesis.^4^ Due to the Warburg effect, cancer cells should present an increased demand of amino acids as alternative fuel sources,^5^ so that a decreased availability should represent a metabolic disadvantage. Likewise, the significant decrease in serum choline observed in probiotics‐receiving groups should affect cancer growth since choline is required for membrane synthesis to support proliferation and for generating second messenger molecules in mitogenic pathways.^6^ Interesting was also the significant decrease of pyruvic acid induced by probiotics, which led us to speculate that probiotics intake could have partially rescued mitochondrial respiration, decreasing Warburg effect. To get more insight into the role of the single probiotic strains included in our blend on PC and gemcitabine effect, BxPC‐3 cells were cultured with probiotic cell‐free supernatants (CFSs), with or without gemcitabine. The preventive effect on EMT was confirmed, especially mediated by *Bifidobacterium bifidum* and *Bifidobacterium breve* (Figure S2), which were also the most effective strains in arresting cell cycle (Figure S3), inducing apoptosis (Figure S4) and which clustered together when the metabolomic profile of the CFSs was analyzed (Figure 4A–D). Interestingly, when investigating the metabolic pathways with the highest impact in probiotic CFSs, some findings (amino acids, pyruvate and butyrate metabolisms) overlapped with the in vivo results (Figure S5). Interestingly, four metabolites resulting as condensation products of tryptophan—tetrahydro β‐carbolines—were found to be discriminant between control broth and probiotic CFSs, namely tetrahydro‐β‐carboline carboxylic acid, methyl tetrahydro‐β‐carboline carboxylic acid (two isomers), and methyl tetrahydro‐β‐carboline‐dicarboxylic acid. They were found enriched especially in *B. breve*, *B. bifidum*, *L. plantarum*, and *L. salivarius* metabolomes (Figure 4E). Their MS2 spectra were compared with analytical standard of 2,3,4,9‐tetrahydro‐1H‐beta‐carboline‐1‐carboxylic acid (C12H12N2O2, MW 216.24), 1,2,3,4‐tetrahydroharmane‐3‐carboxylic acid (C13H14N2O2, MW 230.26), thus annotated at level 2 of confidence. For the dicarboxylic metabolites the similar pattern of fragmentation was found as for the mono carboxylic metabolite (Figure 4F). It is important to note that β‐carboline alkaloids exhibit a broad spectrum of pharmacological properties, including anti‐inflammatory and anticancer activities.^7^, ^8^ More details about CFSs effects are provided in supplementary information. Overall, our results suggest that specific probiotics administration could help relieving some adverse effects of gemcitabine in the treatment of PC by restoring a favorable microbiota. Should these findings be confirmed in the clinical setting, it would represent a milestone in the management of PC patients. Indeed, improving patient's quality of life is a fundamental goal to pursue, since chemotherapy‐related toxicity often requires dose reduction or treatment discontinuation, thereby reducing the chances of cure.

**FIGURE 3:**
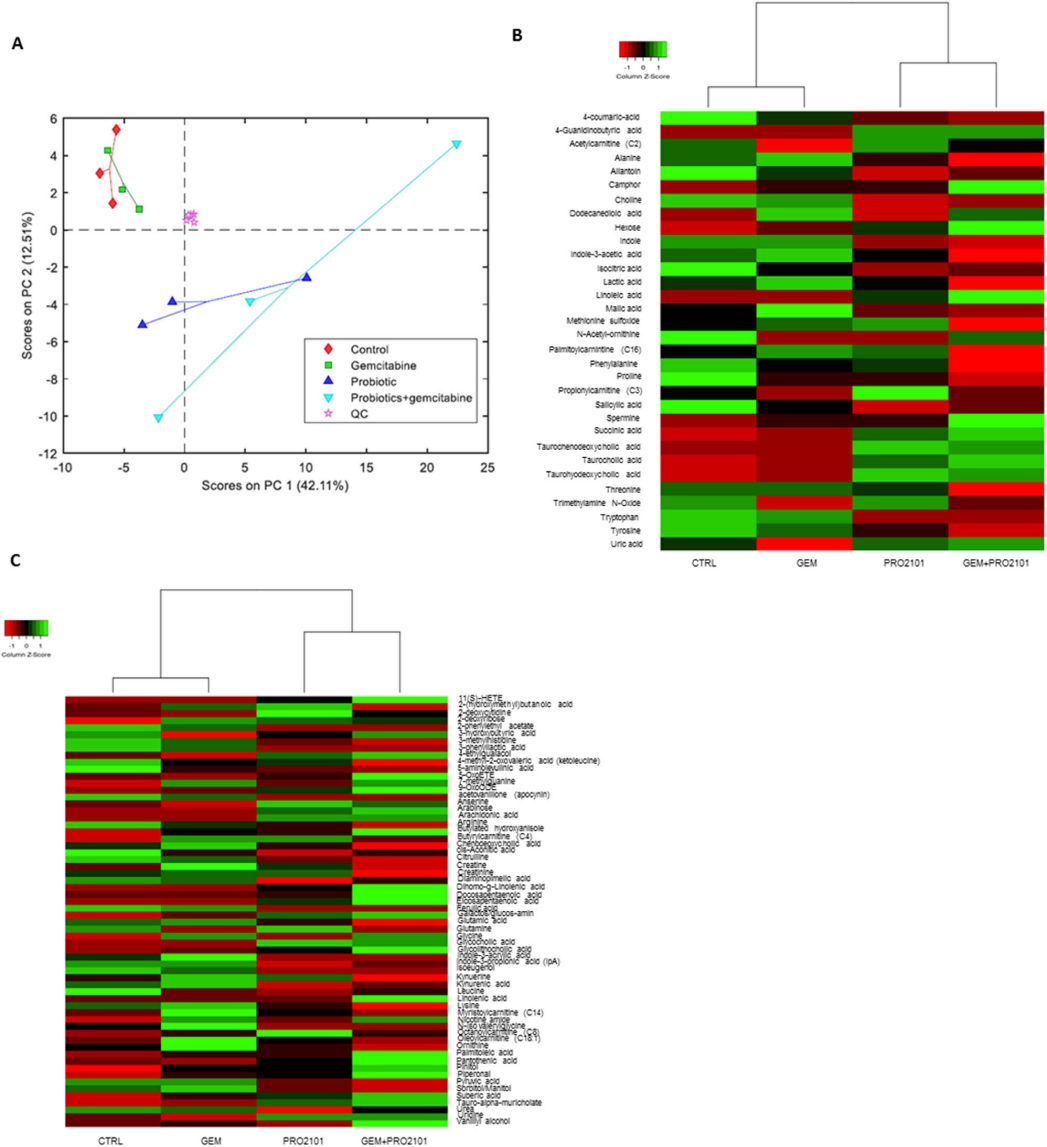
Gemcitabine and/or probiotic treatment impact on serum metabolomics. (A) Score plot from PCA model calculated on the relative concentrations of the serum variables annotated on level 1, 2a, and 2b in the reduced dataset. Data has been autoscaled. Heatmap representation of serum metabolites in 1 (B) and 2a (C) annotation levels, respectively. Data are expressed as means of three mice per experimental group

**FIGURE 4:**
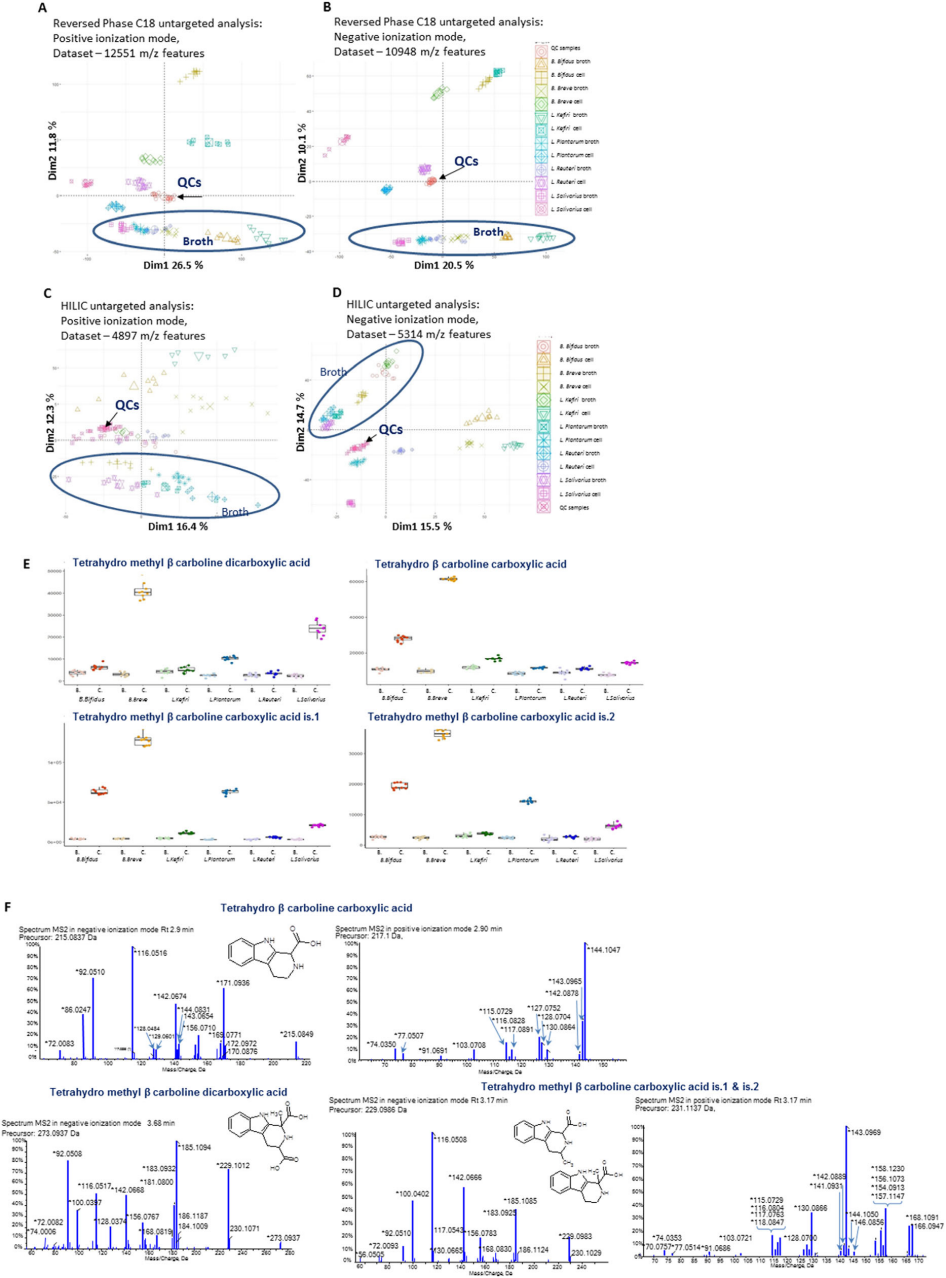
Quality inspection of CFSs metabolomics and tetrahydro β‐carboline compounds. PCA plots of m/z features detected in probiotic CFSs and their control broths for RP– (A), RP+ (B), HILIC– (C), and HILIC+ (D), respectively. Samples indicated with “cell” refer to CFSs, samples indicated with “broth” refer to control MRS broths. Box plots showing the abundance of tetrahydro‐β‐carboline carboxylic acid, methyl‐tetrahydro‐β‐carboline‐dicarboxylic acid and methyl‐tetrahydro‐β‐carboline carboxylic acid isomers in probiotic‐derived CFSs (indicated with C) and their relative control broths (indicated with B) (E). MS2 fragmentation patterns of tetrahydro‐β‐carboline carboxylic acid, methyl‐tetrahydro‐β‐carboline‐dicarboxylic acid and methyl‐tetrahydro‐β‐carboline carboxylic acid isomers (F)

## CONFLICT OF INTEREST

No potential conflicts of interest were disclosed by the other authors.

## Supporting information

Supporting Information

Figure S1

Figure S2

Figure S3

Figure S5

Figure S4

Table S1: Single dose probiotic blend composition

Table S2: Blood count and biochemical parameters

Table S3: Full list of annotated metabolites in probiotic‐derived CFSs and control broths
