# Supplementary material for: Tuning gut microbiota through a probiotic blend in gemcitabine‐treated pancreatic cancer xenografted mice
Source: Clin Transl Med. 2021 Nov 4;11(11):e580. doi: 10.1002/ctm2.580 (PMC8567057; doi:10.1002/ctm2.580)
Supplement: Supplementary file 1 — Supporting Information [file CTM2-11-e580-s002.docx]

**Supplementary information**

**Materials and methods**

**1. Animal experiments**

The study was approved by the Italian Ministry of Health, with the approval number 210/2019-PR.

1 x 10^6^ BxPC-3 human cells were resuspended in 0.1 ml of PBS/matrigel mixture (1:1) and subcutaneously injected in the flank of 5-6weeks old female nude BALB/c mice. The tumors were allowed to grow until an average volume of 100 mm^3^, then the mice were randomly divided into the following four experimental groups: CTRL (control), GEM (25mg/kg/week gemcitabine intraperitoneally), PRO2101 (probiotics mixture by oral gavage five consecutive days/week), GEM+PRO2101 (25mg/kg/week gemcitabine intraperitoneally and pre/prebiotic mixture by oral gavage five consecutive days/week). The probiotic preparation used in this study consisted of six lyophilized probiotic strains (*Bifidobacterium breve* SGB 01, *Bifidobacterium bifidum* SGB 02, *Lactobacillus reuteri* SGL 01, *Lactobacillus salivarius* SGL 03, *Lactobacillus plantarum* SGL 07, *Lactobacillus kefiri* SGL 13), mixed with inulin and lactoferrin. The number of CFUs for each strain and the exact quantities of inulin and lactoferrin contained in a single dose, together with the specific health function, are reported in Table S1. The probiotic doses administered to mice were calculated by converting human dosages according to the formula reported in [1]. Animals had free access to food and water and were monitored daily for sign of illness. Animal body weight and tumor volume was measured weekly. After four weeks, at the end of the treatment, blood was collected from the mandibular plexus, fresh fecal pellets were harvested from the cages and both were stored at -80°C. Upon sacrifice by CO_2_ inhalation, the tumor masses and the intestines were explanted, and both were partly snap-frozen in liquid nitrogen and partly formalin-fixed for histological analyses.

**2. Histological and immunohistochemical analyses**

For histopathological and immunohistochemical analyses frozen formalin-fixed OCT-embedded tissues were cryostat sectioned. Haematoxylin/Eosin (Diapath) staining was performed according to standard protocol for assessing histological features. To visualize stromal fibers in tumor sections Picrosirius Red staining (Scy Tek Lab, SRS-IFU) and Masson’s Trichrome (Diapath, 010210) were performed. For proliferating cells detection, sections were fixed in 100% cold acetone for 2 min, blocked with 2% FBS serum in PBS for 60 min and incubated overnight with anti-Ki67 (1:50, Thermofisher, MAB-14520). To evaluate the extent of DNA double strand breaks, tumor sections were fixed in 80% ethanol for 10 min, blocked with 2% FBS serum in PBS for 60 min and incubated overnight with anti phospho-Histone H2A.X (phospho S139, 1:1000, Abcam, ab11174). To evaluate the expression of an epithelial phenotype, which could be modulated upon mesenchymal transition, tumor sections were fixed in 80% ethanol for 10 min, blocked with 2% FBS serum in PBS for 60 min and incubated overnight with anti E-cadherin (1:100, Cell Signaling, 14472) and anti N-cadherin (1:100, Cell Signaling, 13116). The antibodies were detected using a polymer detection kit (GAM/R-HRP, Microtech) followed by a diaminobenzidine chromogen reaction (Peroxidase substrate kit, DAB, SK-4100; Vector Lab). All sections were counterstained with Harris's Hematoxylin and visualized using a bright-field microscope. Histological visualization of intestinal mucins was performed using Alcian Blue pH 2.5/PAS staining (Bioptica 04-163802).

**3. Immunoblotting**

Protein extraction was obtained from frozen tumors. Samples were homogenized in RIPA buffer with protease inhibitors, as described elsewhere [2]. Bradford assay was performed to quantify protein content. Equal amounts of proteins per sample were separated by SDS-PAGE, then transferred on a PVDF membrane. Membranes were incubated overnight at 4°C with primary antibodies as follows: E-cadherin (1:1000, Cell Signaling Technology, #3195), N-cadherin (1:1000, GeneTex, # GTX101141), Snail (Cell Signaling Technology, #3879), Slug (1:1000, Cell Signaling Technology, #9585), Vimentin (Santa Cruz, #sc-6260), GAPDH (1:20000, Millipore, #A8516). After incubation for 1h at room temperature with HRP-labeled anti-mouse or anti-rabbit IgG (1:3000, BioRad), chemiluminescence was detected by Pierce ECL Plus (ThermoScientific).

**4. Microbiota analysis**

Mouse fecal DNA was extracted using the QIAamp DNA Stool Mini Kit (Qiagen) according to the manufacturer’s instructions to increase the ratio of microbial to host DNA. The V3-V4 hypervariable region of bacterial 16S rRNA gene was amplified using universal primers selected from Klindworth et al. [3]. Dual-indexed libraries were prepared according to the Illumina 16S Metagenomic Sequencing Library Preparation protocol, as previously described [4].

Paired-end sequencing (2 x 300 cycles) was performed on an Illumina MiSeq device. Sequence data generated as FASTQ files, are deposited in the Arrayexpress repository under accession code E-MTAB-10676. De-multiplexed FASTQ files were analyzed using the 16S Metagenomics GAIA 2.0 tool (Sequentia Biotech, Barcelona, Spain, 2017; Benchmark of Gaia 2.0), which performs the quality control of the reads/pairs (i.e., trimming, clipping and adapter removal) through FastQC and BBDuk. The reads/pairs are mapped with BWA-MEM against the custom databases (based on NCBI).

**5. Serum metabolomics**

Serum metabolomic analysis was carried out by MS-Omics as follows. The analysis was carried out using a Thermo Scientific Vanquish LC coupled to Thermo Q Exactive HF MS. An electrospray ionization interface was used as ionization source. Analysis was performed in negative and positive ionization mode. The UPLC was performed using a slightly modified version of the protocol described by Doneanu et al. [5]. Peak areas were extracted using Compound Discoverer 2.0 (Thermo Scientific). Identification of compounds was performed at four levels; Level 1: identification by retention times (compared against in-house authentic standards), accurate mass (within 3ppm error), and MS/MS spectra, Level 2a: identification by retention times (compared against in-house authentic standards), accurate mass (within 3 ppm error). Level 2b: identification by accurate mass (within 3 ppm error), and MS/MS spectra, Level 3: identification by accurate mass alone (within 3 ppm error).

**6. Probiotic cell-free supernatants production**

In order to prepare cell-free supernatants (CFSs), each probiotic strain was grown and expanded in MRS broth (BD Difco Laboratories, Detroit, MI, USA) for 24-48h, depending on the strain, at 37°C under anaerobic conditions using AnaeroGen bags (Thermo Scientific, Waltham, Massachusetts, USAOxoid). Briefly, CFSs were obtained by centrifuging the bacterial cultures (3.000 xg, 10 minutes), followed by filtration of the obtained supernatant through a 0.22 μm pore size filter (Sartorius, Goettingen, Germany). Then, CFSs were supplemented with 1% penicillin/streptomycin (Sigma-Aldrich, St. Louis, MO) and stored at -20°C until use.

**7. BxPC-3 cell culture, co-culture with probiotic CFS and gemcitabine treatment**

BxPC-3 cells were cultured in RPMI 1640 medium supplemented with 10% fetal bovine serum (FBS), 100 U/ml penicillin and 100 μg/ml streptomycin (Invitrogen Life Technologies, Milan, Italy) in 5% CO2 atmosphere at 37°C.

For co-culture experiments with probiotic-derived CFSs, BxPC-3 cells were plated onto 12-well plates. After 24 hours, CFSs from the six probiotic strains or MRS as control were administered to cells at a concentration of 20% (v/v), with or without 1µM gemcitabine for 48 hours.

**8. Scratch assay**

BxPC-3 grown at confluence into 12-well plates were subjected to a scratch in the monolayer performed with a p200 pipette tip, then were washed with 1X PBS to remove cell debris before adding RPMI supplemented with MRS or CFS in the presence or absence of gemcitabine for 48h as described above. Cell migration was monitored by taking pictures at 0h, 24h and 48h post treatment. The scratch area at each time-point was measured with Image J software.

**9. Cell cycle analysis**

BxPC-3 cells treated as described above were harvested by trypsinization, resuspended in complete RPMI then fixed with 1ml of 70% cold ethanol following the Muse Cell Cycle Kit instructions (Luminex, Austin, Texas) at -20°C for at least three hours before the assay. Ethanol-fixed cells were incubated with the premixed reagent containing propidium iodide and RNAse A for 30 minutes at room temperature in the dark, according to the supplied staining protocol of the kit, before loading on Muse Cell Analyzer (Millipore, Italy).

**10. Apoptosis assay**

BxPC-3 cells treated as described above underwent quantification of live, early apoptotic, late apoptotic and dead cells at the Muse Cell analyzer by using the Muse Annexin V and Dead Cell Assay kit (Luminex, Austin, Texas), accordingly to the supplier’s instructions. Briefly, a cell suspension was incubated with an equal volume of Muse Annexin V & Dead Cell Reagent for 20 minutes in the dark, before loading on the Muse Cell Analyzer.

**11. Metabolomic analysis of CFSs**

CFS containing excreted metabolites and control broth samples were extracted for untargeted mass spectrometry metabolomics profiling. First step consisted of normalization between samples for CPU /ml value by dilution with MilliQ water to obtain the 2.67*10^5^ CFU in 300 µl. After that, samples were extracted in biological triplicates, and analyzed in HILIC and Reversed Phase fashion, following the protocol by Drago et al. [6]. Briefly, for RP modality analysis, 30 µl of sample were transferred into new Eppendorf tube and 100 µL of cold extraction buffer was added (39% AcN/61% H_2_O). For HILIC analysis modality, 30 µl of samples were placed into Eppendorf tube and 100 µl of cold extraction buffer was added (100% AcN). Such prepared samples were injected on Triple TOF 5600+ mass spectrometer on two chromatographic columns: Waters UPLC Acquity HSS T3, 1.8 µm; 2.1 x 100 mm and Waters UPLC BEH Amide 1.7 µm; 2.1x150 mm, for RP and HILIC modalities respectively. The injection volume was 5 µl.

Triple TOF 5600+ mass spectrometer (SCIEX) coupled to the UPLC 1290 system (Agilent Technologies), was operating under positive and negative ionization modes with SWATH data acquisition. The RP chromatographic conditions were adopted from Want et al. [7]. Briefly, flow rate was set at 0.6 ml/min with a gradient of solvent A (water, 0.1% formic acid) and solvent B (methanol, 0.1% formic acid). The gradient, in both the positive and negative mode started from 2% of B; increased up to 95% of B in 14 min; maintained constant at 95% of B for 5 min and decreased to 2% of B in 1 min. The column was set at 50°C while the samples were kept at 4°C. BEH Amide HILIC gradient was adopted from Paglia et al. [8]. Briefly, the flow rate was set at 0.6 ml/min with a gradient of solvent A (acetonitrile, 0.1% formic acid) and solvent B (water, 0.1% formic acid). The gradient, in both positive and negative mode, started from 2% of B; increased up to 60% of B in 10 min; maintained constant at 60% of B for 2 min and decreased to 2% of B in 1 min. The column was set at 40°C while the samples were kept at 4°C.

Mass range for RP analysis was 50-850 m/z and for HILIC 50-500 m/z. For RP modality, the TOF-MS survey was set to 95 ms, while SWATH to 60 ms, number of SWATH windows were 16, each of 50Da covering 50-850 m/z range. In HILIC modality TOF-MS survey was set to 150 ms, while SWATH to 70 ms, SWATH windows were 10, each of 45 Da covering 50-500 mass range.

The ion source-dependent parameters were optimized for positive (and negative) modes: ion spray voltage floating (ISVF) 5500V (-4500); Declustering Potential (DP): 80 (-80); Collision Energy (CE): 10 (-10); temperature 550°C; curtain gas, 25 (arbitrary units) and ion source gas (GS1 and GS2) at 40 psi. For SWATH fragmentation CE ± CES were 35 ± 15 and -35 ± 15 for positive and negative ionization modes respectively. Automated calibration was performed using an external calibrant delivery system (CDS) which infuses APCI positive or negative calibration solution every 5 samples injection.

The queue consisted of 157 injections, with fully randomized samples and double QC injection every 8 study samples. The raw .wiff files were converted in .abf with Reifycs Abf Converter (https://www.reifycs.com/AbfConverter), and analysed with MS-DIAL (version 4.48) separately for negative and positive ionization modes for each chromatographical column (RP+, RP-, HILIC+, HILIC-), following the workflow published elsewhere [9]. Four datasets were saved as .txt from MS-DIAL and further data analysis was performed in R, following data-processing according to Garcia-Aloy et al [10]. Annotation was performed against in house spectral library containing over 700 metabolites, and whenever necessary a manual structure elucidation. Level of annotations were reported in paragraph 3.7. A Metabolite Set Enrichment Analysis (MSEA) was performed through MetaboAnalyst on-line engine [11, 12]. The 99 HMDB codes were used as input data, while the Homo sapiens KEGG library was used as database.

**12. Statistics**

The results are shown as mean ± standard deviation. Student t-test was used to compare mean in pairwise comparisons. Results were considered significant when *p*<0.05(*), *p*<0.01(**), *p*<0.001(***). In metabolomics assay for CFSs samples, the ANOVA was performed on entire dataset. Additionally, t-test and FDR corrections were performed for each pair: CFS and corresponding broth, together with calculation of absolute fold change.

**Results**

**13. Effect of gemcitabine and probiotics administration on tumor epithelial-mesenchymal transition**

Immunohistochemical evaluation of E-cadherin and N-cadherin expression, epithelial and mesenchymal markers respectively, as depicted in Figure S1A, showed for E-cadherin a diffuse though variably intense staining with N-cadherin marking areas densely populated by spindle-shaped cells in control tissues. In gemcitabine group, E-cadherin staining was less diffuse and dense and N-cadherin was multifocally induced, supporting a lower expression of an epithelial phenotype consistent with the morphological switch observed on H&E. Upon probiotic treatment, E-cadherin showed a strong and diffuse staining and N-cadherin was globally down-modulated, whereas in the combined treatment, the E-cadherin staining was reduced in foci with an epithelial-mesenchymal transition (EMT) morphology, immunoreactive for N-cadherin. The EMT was further investigated by immunoblot on tumor protein extracts, by assessing the expression of the mesenchymal markers Snail, Slug and Vimentin, beside that of E- and N-cadherin. Consistently with IHC results, a milder mesenchymal phenotype was observed upon probiotics administration, with N-cadherin significantly reduced compared to control group (*p*=0.049). Moreover, probiotics combined to gemcitabine, with respect to gemcitabine alone, produced a remarkable decrease in mesenchymal markers, with N-cadherin, Snail and Vimentin reaching statistical significance (*p*=0.039, *p*=0.046, *p*=0.011, respectively) (Figure S1B-C).

**14. Gemcitabine and probiotic treatments affect gut microbiota composition, structure and function**

The composition of the bacterial communities at the phylum, family, genus and species level in the four treatment groups is represented in Figures 2C-F, respectively. At the phylum level, no major variation among the groups was detected, with *Firmicutes* and *Bacteroidetes* being the most represented microorganism in all four experimental conditions. At the family level, gemcitabine treatment in comparison to control produced a significant decrease in *Bacteroidaceae* (0.42% *vs* 0.63%, *p*=0.049), *Desulfohalobiaceae* (0% *vs* 0.012%, *p*=0.010), *Moraxellaceae* (0.024% *vs* 0.032%, *p*=0.025), *Prolixibacteriaceae* (0% *vs* 0.017 %, *p*=0.016), *Sutterellaceae* (0.014% *vs* 0.028%, *p*=0.009) and *Tissierellaceae* (0% *vs* 0.024%, *p*=0.0005), whereas a *Colwelliaceae* (0.012% *vs* 0%, *p*=0.0009) and *Legionellaceae* (0.010% *vs* 0 %, *p*=0.0009) significantly increased. Similar to gemcitabine alone, also in gemcitabine+probiotics treatment *Desulfohalobiaceae* and *Prolixibacteriaceae* remained undetected, while *Legionellaceae,* absent in control and probiotic groups, were still found in co-treatment (0.010%). On the contrary, the combined treatment significantly restored *Tissierellaceae* abundance canceled by gemcitabine (0.026% vs 0%, *p*=0.030). Other significant changes promoted by double treatment compared to gemcitabine were the under-representation of *Clostridiaceae* (9.2% *vs* 13.9%, *p*=0.044) and *Colwelliaceae* (0% *vs* 0.012%, p=0.0009), and the over-representation of *Comamonadaceae* (0.018% *vs* 0%, *p*=0.047), *Desulfomicrobiaceae* (0.016% *vs* 0%, *p*=0.010), *Eubacteriaceae* (butyrate-producers) (5.6% *vs* 3.2%, *p*=0.038) and *Hyphomonadaceae* (0.011% *vs* 0%, *p*=0.037). Worthnote was also the family of *Phyllobacteriaceae*, detected only in probiotics group (0.014%) and absent in the others. Among the genera, gemcitabine treatment compared to control caused a drop in *Bacteroides* (0.39% *vs* 0.60%, *p*=0.043), *Bittarella* (0.014% *vs* 0.051%, *p*=0.001), *Parasutterella* (0.011% *vs* 0.027%, *p*=0.012), *Prolixibacter* (0% *vs* 0.013%, *p*=0.011), *Psychrobacter* (0.020% *vs* 0.029%, *p*=0.020), *Ruthenibacterium* (0% *vs* 0.019%, *p*=0.014) and *Tissierella* (0% *vs* 0.015%, *p*=0.012), increasing *Litorilituus* (0.011% *vs* 0%, *p*=0.012), *Massilimaliae* (0.019% *vs* 0.010%, *p*=0.011) and *Propionibacterium* (1.13% *vs* 0.14%, p=0.043) abundance instead. Combining gemcitabine treatment with probiotics restored normal abundance of *Bittarella* (0.044% *vs* 0.014%, *p*=0.0006), *Ruthenibacterium* (butyrate producer) (0.015% *vs* 0%, *p*=0.027) and *Litorilituus* (0. % *vs* 0.011%, *p*=0.012) which were altered by chemotherapy alone. In addition, *Carboxylicivirga* (0.011% *vs* 0%, *p*=0.013), *Desulfomicrobium* (0.010% *vs* 0%, *p*=0.010), *Desulfotomaculum* (0.012% *vs* 0%, *p*=0.005), *Desulfovibrio* (0.015% *vs* 0%, *p*=0.002), *Erysipelothrix* (0.014% *vs* 0%, *p*=0.037), *Faecalicatena* (SCFA producer) (0.025% *vs* 0%, *p*=0.045) and *Lactobacillus* (generally regarded as beneficial bacteria) (0.25% *vs* 0.18%, *p*=0.034) were repleted, while *Catonella* (0% *vs* 0.011%, *p*=0.046) and *Clostridium* (8.5% *vs* 12.6%, *p*=0.050) were reduced by gemcitabine+probiotics versus gemcitabine treatment. A number of bacterial genera, moreover, were modified when administering probiotics alone in comparison with control. This was the case of *Anaerobacterium* (0.020% *vs* 0.036%, *p*=0.025), *Bittarella* (0% *vs* 0.051%, *p*=0.0007), *Papillibacter* (0% *vs* 0.022%, *p*=0.036) and *Prolixibacter* (0% *vs* 0.011%, *p*=0.046) which were depleted and of *Geofilum* (0.019% *vs* 0%, *p*=0.009), *Litorilituus* (0.010% *vs* 0%, *p*=0.015), *Marseillibacter* (0.011% *vs* 0%, *p*=0.006), *Mesorhyzobium* (0.014% *vs* 0%, *p*=0.014), *Pseudobutyrivibrio* (butyrate producer) (0.048% *vs* 0.015%, *p*=0.042) and *Wigglesworthia* (0.022% *vs* 0.014%, *p*=0.012) which resulted, on the contrary, over-represented.

Several differences emerged at species level, among which *Clostridium methylpentosum*, *Marvinbryantia formatexigens* and *Robinsoniella peoriensis* significantly decreased upon gemcitabine treatment compared to untreated control (0.013% *vs* 0%, *p*=0.0001; 0. 20% *vs* 0.10%, *p*=0.040; 0.028% *vs* 0.014%, *p*=0.017, respectively), but were restored to some extent when gemcitabine was supplemented with probiotics (0.012%, *p*=0.18 for *C. methylpentosum*; 0.22%, *p*=0.26 for *M. formatexigens*; 0.023%, *p*=0.017 for *R. peoriensis*). Further significant differences between GEM + PRO2101 versus GEM included the increased abundance of *Blautia producta* (0.047% *vs* 0.032%, *p*=0.030) and *Clostridium indolis* (0.017% *vs* 0%, *p*=0.0005), which are butyrate-producing bacteria. Finally, among the species significantly altered by probiotics treatment compared to control, a decrease in *Clostridium cocleatum* (0.024% *vs* 0.046%, *p*=0.002) and an increase in *Colidextribacter massiliensis* (0.012% *vs* 0%, *p*=0.005) and *Roseburia sp. 499* (butyrate producer) (0.010% *vs* 0%, *p*=0.039) were registered.

Besides all these gut microbes significantly changed in response to treatments, worthnote is the differential abundance of a number of taxa which, though not reaching statistical significance, tended to be enriched in mice receiving probiotics, alone or in combination with chemotherapy. Among these, *Defluviitaleaceae* (0.014% in PRO2101 *vs* 0% in CTRL, 0.017% in GEM+ RO2101 *vs* 0% in GEM)*,* *Faecalibacterium* (0.25% in PRO2101 *vs* 0.17% in CTRL, 0.14% in GEM+PRO2101 *vs* 0.06% in GEM), *Flintibacter* (0.26% in PRO2101 *vs* 0% in CTRL, 0.37% in GEM+PRO2101 *vs* 0% in GEM) and its related species *Flintibacter butyricus* (0.22% in PRO2101 *vs* 0% in CTRL, 0.25% in GEM+PRO2101 *vs* 0% in GEM), *Roseburia hominis* (0.024% in GEM+PRO2101 *vs* 0 % in GEM) which, together with other aforementioned bacteria enriched in the same groups, are known producers of butyrate and other SCFAs endowed with anti-inflammatory and health beneficial properties for the host.

**15. Hematological and biochemical parameters’ assessment**

With the aim to evaluate the effect of probiotics on any toxicity due to chemotherapy, hematological and biochemical parameters were assessed in all mice at the end of the treatment. Mean values for blood count and serum renal and hepatic parameters in each experimental group are reported in Table S2. Strikingly, all but one mice in GEM group exhibited a number of red blood cells below the reference interval, whereas none of the animals in the GEM+PRO2101 group did the same. This resulted in a mean value of red blood cells which was lower, although not reaching statistical significance, in GEM versus GEM+PRO2101 group. Gemcitabine administration also caused a significant increase in the percentage of Red Blood Cell Distribution Width (RDW) compared to control. Mean Corpuscolar Volume (MCV) and Mean Corpuscolar Hemoglobin (MCH) were also significantly lower in combined treatment versus gemcitabine alone while falling within normal values in both groups.

Intriguingly, a marked decreasing trend in platelet count was found in GEM mice with respect to CTRL. Combining gemcitabine with probiotics resulted in statistically significant recovery of platelet number and statistically significant increase in plateletcrit compared to gemcitabine alone. Serum biochemical parameters were also assessed, as summarized in Table S2. No significant difference was observed for hepatic markers, which all felt within the reference range, although a decreasing trend for aspartate aminotransferase (AST) and gamma-glutamyltransferase (GGT) was measured in animals receiving combined treatment relative to those receiving gemcitabine alone. As for renal parameters, instead, urea and creatinine levels were above the reference range in all mice, suggesting a renal damage likely due to cancer. However, a slight though not significant decrease upon probiotics administration was recorded for both markers.

**16. Effect of probiotic cell-free supernatants on pancreatic cancer cells migration, cell cycle and apoptosis**

Based on the concept that each probiotic bacterium has its own specific characteristics and mechanisms of action, to get more insight into the role of the single six probiotic strains included in our blend on pancreatic cancer and gemcitabine effect, BxPC-3 cells were cultured *in vitro* with probiotic cell-free supernatants in the presence or in the absence of gemcitabine.

The scratch assay revealed that, compared to the MRS control, *B. breve* and *B. bifidum* were the most effective strains in inhibiting cell migration, since the (Figure S2A and S2C) and in the presence (Figure S2B and S2D) of gemcitabine. A less pronounced but statistically significant inhibition of scratch closure was also observed upon *L. salivarius* treatment after 24h, either in the presence and in the absence of gemcitabine and upon *L. kefiri* and gemcitabine co-incubation for 24 and 48 h. To dissect the impact of probiotics on cancer cell growth, cell cycle and apoptosis were investigated. As shown in Figure S3, CFSs induced many variations in the distribution of the cell cycle phases compared to MRS control, in the absence and in the presence of gemcitabine. In detail, the percentage of cells in G0/G1 phase decreased while concomitantly that in G2/M increased upon *B. breve*, *B. bifidum*, *L. kefiri* and *L. salivarius* treatment, with respect to MRS control. In addition, *B. bifidum* also augmented cells in S phase relative to MRS. Regarding CFSs + gemcitabine co-treatments, cells incubated with both *B. breve +* GEM and *B. bifidum +* GEM showed a decrease in G0/G1 phase and concomitant increases in S and G2/M phases, with respect to MRS + Gem. Similarly, in cells treated with *L. kefiri* + Gem, the decrease in G0/G1 phase was accompanied by an increase in G2/M phase, whereas in *L. salivarius* + GEM, the decrease in G0/G1 phase was compensated by an increase in S phase, when compared to MRS + Gem. Finally, a slight but significant decrease in G0/G1 phase was also observed in cells incubated with *L. reuteri* + GEM, without any other change in S nor in G2/M phases. We next evaluated whether probiotics could induce apoptosis in pancreatic cancer cells. As reported in Figure S4, both *B. breve* and *B. bifidum* caused a drop in the percentage of live cells and a parallel strong increase in late and total apoptotic cells. Less striking but still significant were the decrease in live cells and the rise in early, late and total apoptotic cells induced by *L. kefiri* and *L. salivarius*. Finally, a slight increase in early apoptotic cells without any other significant change was observed upon *L. plantarum* and *L. reuteri* CFS incubation. Concerning CFSs + gemcitabine co-treatments, again *B. breve +* GEM and *B. bifidum +* GEM strongly promoted apoptosis, as compared to MRS + GEM condition. Among other combined treatments, only *L. salivarius* + GEM caused significant variations in the percentage of live, late and total apoptotic cells with respect to MRS + GEM.

**17. Metabolomics analysis of cell-free supernatants**

The untargeted metabolomics of CFSs was executed to investigate the excreted signaling molecules in single probiotic strains, and interpret them in light of potentially protective characteristics observed in cancer cells migration, cell cycle and apoptosis experiments**.** Numbers of *m/z* features detected for each column and polarity were: 10276, 17338, 5650, 6775 for RP-, RP+, HILIC- and HILIC+, respectively. Subtraction of blank samples reduced the number of around 5%. Data inspection was performed through visualization of each dataset with PCA, verification of mass accuracy and retention time stability over the entire queue. The four PCA plots are reported in Figure 4A-D. The number of *m/z* features reaching statistical significance with ANOVA were: 5190, 11062, 4406, 2714 for RP-, RP+, HILIC- and HILIC+, respectively. For further annotation step, *m/z* features characterized by adjusted *p*-value (with standard Bonferroni FDR correction) < 0.05 and absolute fold change > 5 were selected. Some compounds were automatically annotated thank to the incorporated MS-DIAL libraries, several metabolites were annotated thank to in-house library, while the others underwent manual structure elucidation. A total of 139 metabolites were annotated and classified in different families according to their structure: amino acids, fatty acids, tryptophan condensation products, bi- and tri-peptides, small organic acids. Full list of metabolites with HMDB and KEGG codes is available in Table S3. All metabolites being statistically significant with adjusted *p* value < 0.05 are presented in the clustering heatmap in Figure S5A. The metabolomic profiles of *B. breve*, *B. bifidum* and *L. kefiri* tended to cluster together and the same did *L. salivarius*, *L. plantarum* and *L. reuteri.* Among the most striking results, many dipeptides were found decreased in probiotic CFSs as compared to control broths, whereas many amino acids were enriched in *B. breve*, *B. bifidum* and *L. kefiri* CFSs and depleted in *L. salivarius*, *L. plantarum* and *L. reuteri* CFSs*.*

Interestingly, four metabolites resulting as condensation products of tryptophan – tetrahydro β-carbolines- were found to be discriminant between control broth and probiotic CFSs, namely tetrahydro-β-carboline carboxylic acid, methyl tetrahydro-β-carboline carboxylic acid (two isomers) and methyl tetrahydro-β-carboline-dicarboxylic acid. They were found enriched especially in *B. breve, B. bifidum, L. plantarum* and *L. salivarius* metabolomes (Figure 4E). The origin and synthesis of the tetrahydro-β-carbolines are well described in plants, however their production by microorganisms remains poorly understood. Ghandi et al. proposed mechanism of tetrahydro-β-carboline production through methylglyoxal synthase gene (mgsA) expression. They observed the presence of tetrahydro-β-carboline metabolites in cheese as a result of microbial production (*Lactobacillus casei*) of methylglyoxal that reacted with amino acids [13]. Among other metabolites that exhibit similar trends for *B.* *bifidum* and *B*. *breve* against other CFSs were taurocholic acid (down-regulated) and aconitic acid (up-regulated). Noteworthy, the CFSs from *B. breve* and *B. bifidum* were found enriched in genistein and daidzein, two soy isoflavones endowed with anticancer properties” [14]. Subsequently, an enrichment analysis was performed to identify pathways and biological functions with the highest impact in CFSs metabolomics dataset. The MSEA results represented in Figure S5B revealed that the pathways mostly represented included amino acids, nitrogenous bases and vitamins metabolism.

**References**

1. Reagan-Shaw S, Nihal M, Ahmad N: **Dose translation from animal to human studies revisited.** *FASEB J* 2008, 22(3):659-61.
2. Panebianco C, Adamberg K, Jaagura M, Copetti M, Fontana A, Adamberg S, Kolk K, Vilu R, Andriulli A, Pazienza V: **Influence of gemcitabine chemotherapy on the microbiota of pancreatic cancer xenografted mice.** *Cancer Chemother Pharmacol* 2018, **81:**773-782.
3. Klindworth A, Pruesse E, Schweer T, Peplies J, Quast C, Horn M, Glockner FO: **Evaluation of general 16S ribosomal RNA gene PCR primers for classical and next-generation sequencing-based diversity studies.** *Nucleic Acids Res* 2013, **41:**e1.
4. Picchianti-Diamanti A, Panebianco C, Salemi S, Sorgi ML, Di Rosa R, Tropea A, Sgrulletti M, Salerno G, Terracciano F, D'Amelio R, et al: **Analysis of Gut Microbiota in Rheumatoid Arthritis Patients: Disease-Related Dysbiosis and Modifications Induced by Etanercept.** *Int J Mol Sci* 2018, **19**.
5. Doneanu CE, Chen W, Mazzeo JR: **UPLC/MS Monitoring of Water-Soluble Vitamin Bs in Cell Culture Media in Minutes.** vol. Library Number: APNT134636355 Waters; 2011.
6. Drago D, Andolfo A, Mosca E, Orro A, Nocera L, Cucchiara V, Bellone M, Montorsi F, Briganti A: **A novel expressed prostatic secretion (EPS)-urine metabolomic signature for the diagnosis of clinically significant prostate cancer.** *Cancer Biol Med* 2021.
7. Want EJ, Masson P, Michopoulos F, Wilson ID, Theodoridis G, Plumb RS, Shockcor J, Loftus N, Holmes E, Nicholson JK: **Global metabolic profiling of animal and human tissues via UPLC-MS.** *Nat Protoc* 2013, **8:**17-32.
8. Paglia G, Hrafnsdottir S, Magnusdottir M, Fleming RM, Thorlacius S, Palsson BO, Thiele I: **Monitoring metabolites consumption and secretion in cultured cells using ultra-performance liquid chromatography quadrupole-time of flight mass spectrometry (UPLC-Q-ToF-MS).** *Anal Bioanal Chem* 2012, **402:**1183-1198.
9. Klavus A, Kokla M, Noerman S, Koistinen VM, Tuomainen M, Zarei I, Meuronen T, Hakkinen MR, Rummukainen S, Farizah Babu A, et al: **"notame": Workflow for Non-Targeted LC-MS Metabolic Profiling.** *Metabolites* 2020, **10**.
10. Garcia-Aloy M, Ulaszewska M, Franceschi P, Estruel-Amades S, Weinert CH, Tor-Roca A, Urpi-Sarda M, Mattivi F, Andres-Lacueva C: **Discovery of Intake Biomarkers of Lentils, Chickpeas, and White Beans by Untargeted LC-MS Metabolomics in Serum and Urine.** *Mol Nutr Food Res* 2020, **64:**e1901137.
11. Pang Z, Chong J, Li S, Xia J: **MetaboAnalystR 3.0: Toward an Optimized Workflow for Global Metabolomics.** *Metabolites* 2020, **10**.
12. Xia J, Mandal R, Sinelnikov IV, Broadhurst D, Wishart DS: **MetaboAnalyst 2.0--a comprehensive server for metabolomic data analysis.** *Nucleic Acids Res* 2012, **40:**W127-133.
13. Gandhi NN, Barrett-Wilt G, Steele JL, Rankin SA: **Lactobacillus casei expressing methylglyoxal synthase causes browning and heterocyclic amine formation in Parmesan cheese extract.** *J Dairy Sci* 2019, **102:**100-112.
14. Adjakly M, Ngollo M, Boiteux J, Bignon Y, Guy L, Bernard-Gallon D. **Genistein and daidzein: different molecular effects on prostate cancer.** Anticancer Res 2013, 33(1):39-44.

**Supplementary Figure Legends**

**Figure S1. Impact of gemcitabine and/or probiotic treatment on epithelial-mesenchymal transition**

(A) Immunohistochemical stain of tumors tissues with epithelial-mesenchymal transition markers E-cadherin and N-cadherin. Pictures were taken at 20X magnification. Scale bar is 150µm. (B) Immunoblot analysis of EMT markers’ expression in tumors and (C) radar chart representation of their quantitative analysis.

**Figure S2. Probiotic CFSs’ effect on pancreatic cancer cells migration.** Representative pictures of scratch closure after 24 and 48 h of CFSs treatment in the absence (A) or in the presence (B) of gemcitabine. Percentage of wound closure after 24 and 48 h of CFSs treatment in the absence (A) or in the presence (B) of gemcitabine. Data are expressed as means ± SD of four fields. Differences were considered significant when *p* < 0.05 (*), *p* < 0.01 (**) or *p* < 0.001 (***).

**Figure S3. Probiotic CFSs impact on cell cycle.** Representative plots and relative quantification of cell cycle progression after 48h of treatment, in the absence or in the presence of gemcitabine. Data are expressed as means ± SD of three independent experiments. Differences were considered significant versus the non gemcitabine-treated counterpart when *p* < 0.05 (*), *p* < 0.01 (**) or *p* < 0.001 (***). Differences were considered significant versus MRS when *p* < 0.05 (#), *p* < 0.01 (##) or *p* < 0.001 (###). Differences were considered significant versus MRS + GEM when *p* < 0.05 (†), *p* < 0.01 (††) or *p* < 0.001 (†††).

**Figure S4.** **Probiotic CFSs’ effect on apoptosis.** Representative plots and relative quantification of live, early apoptotic, late apoptotic and total apoptotic cells after 48h of treatment, in the absence or in the presence of gemcitabine. Data are expressed as means ± SD of three independent experiments. Differences were considered significant versus the non gemcitabine-treated counterpart when *p* < 0.05 (*), *p* < 0.01 (**) or *p* < 0.001 (***). Differences were considered significant versus MRS when *p* < 0.05 (#), *p* < 0.01 (##) or *p* < 0.001 (###). Differences were considered significant versus MRS + GEM when p < 0.05 (†), p < 0.01 (††) or p < 0.001 (†††).

**Figure S5. Metabolomic profile of CFSs.** Heatmap representation of metabolites being statistically significant with adj p value < 0.05 among the different CFSs. Each column represents a biological replicate including three technical replicates. Samples indicated with “cell” refer to CFSs, samples indicated with “broth” refer to control MRS broth (A). Enrichment analysis showing the biochemical pathways with higher impact within the statistically significant metabolites in CFSs. Color gradient corresponds to the –log10 (p value) with red being the highest and circle size is proportional to the enrichment ratio (B).

**Table S1:** Single dose probiotic blend composition

**Table S2:** Blood count and biochemical parameters

**Table S3:** Full list of annotated metabolites in probiotic-derived CFSs and control broths.
