# Supplementary figures and images for: Tuning gut microbiota through a probiotic blend in gemcitabine‐treated pancreatic cancer xenografted mice
Source: Clin Transl Med. 2021 Nov 4;11(11):e580. doi: 10.1002/ctm2.580 (PMC8567057; doi:10.1002/ctm2.580)

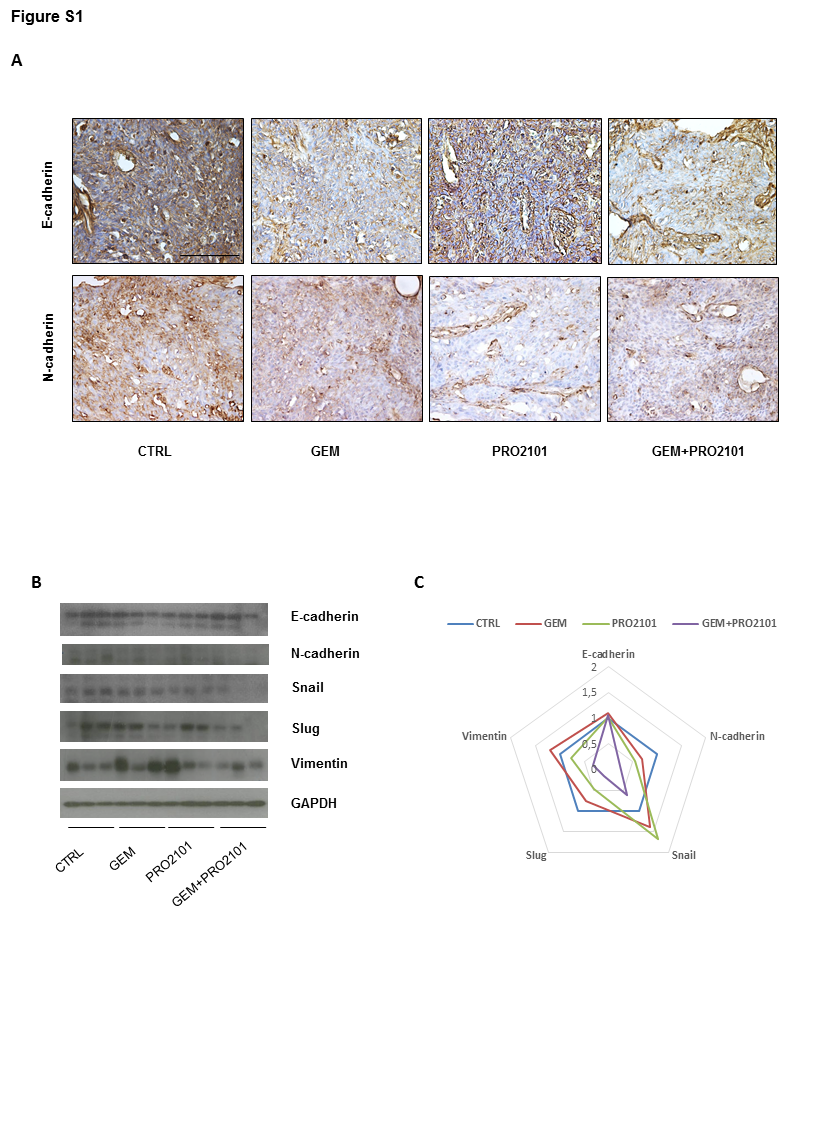

Supplement: Supplementary file 2 — Figure S1 [file CTM2-11-e580-s004.TIF]

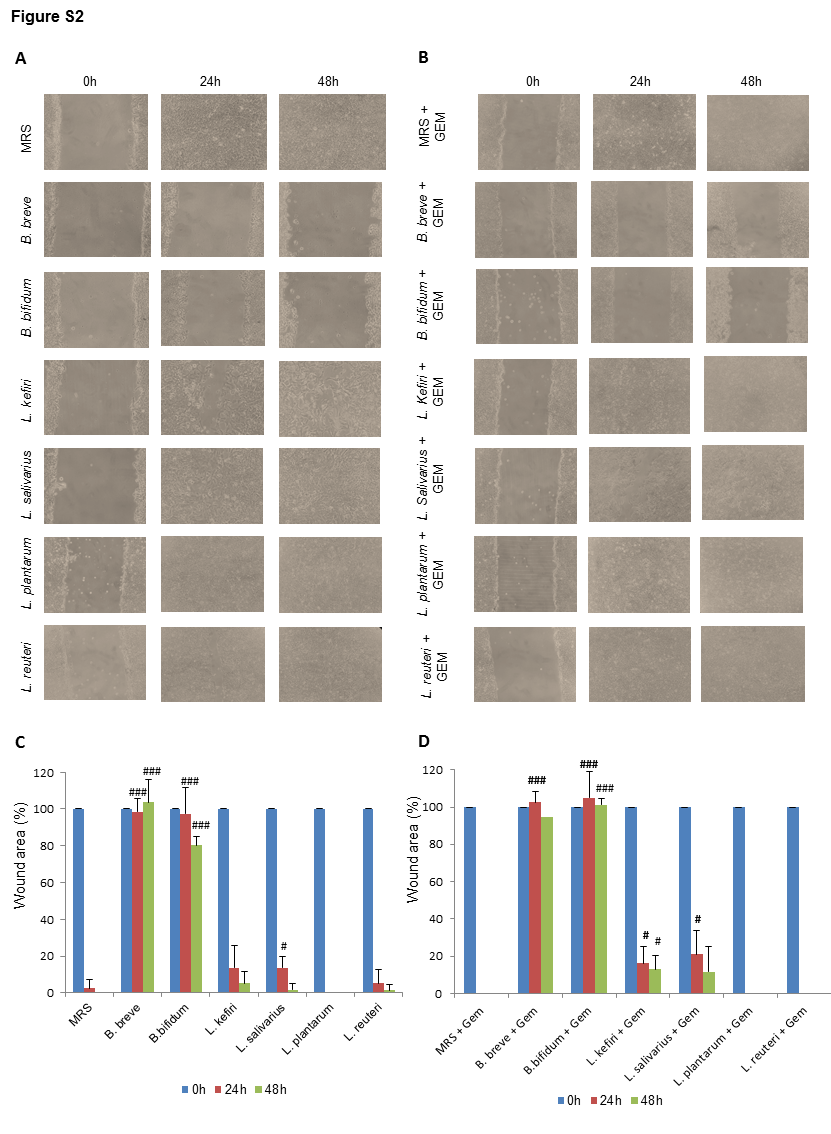

Supplement: Supplementary file 3 — Figure S2 [file CTM2-11-e580-s007.TIF]

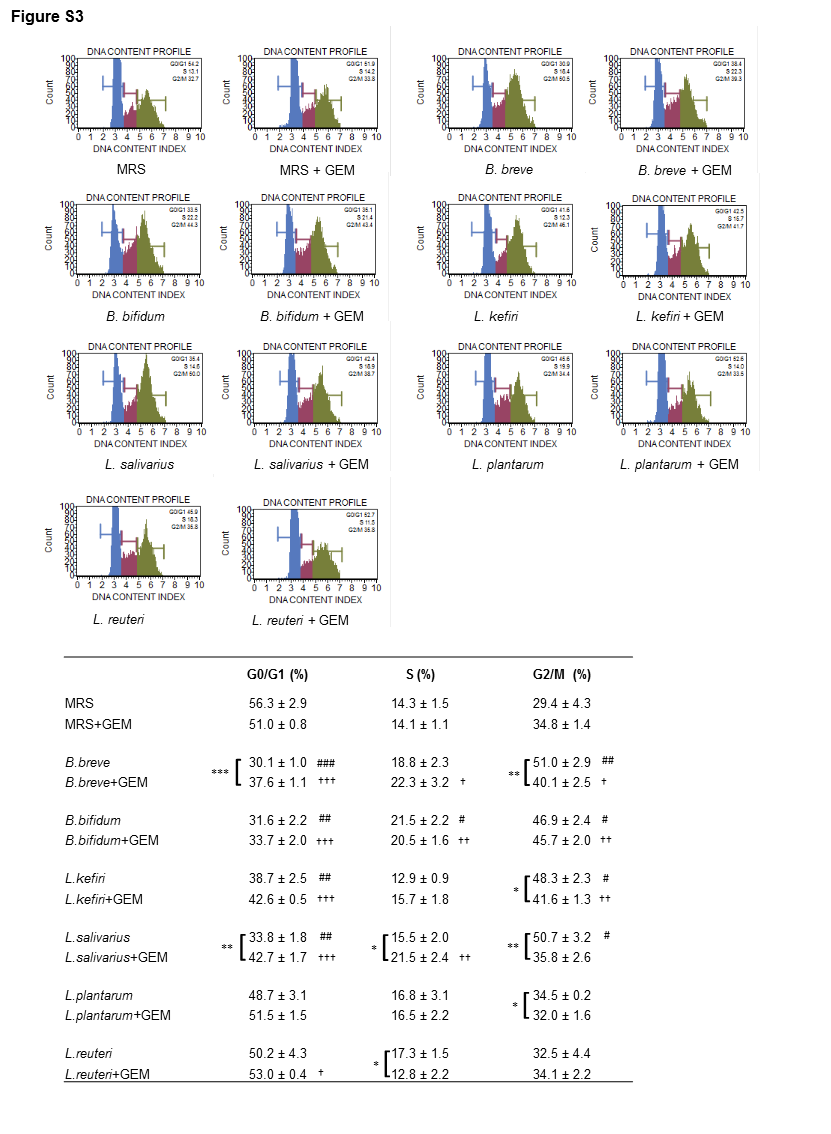

Supplement: Supplementary file 4 — Figure S3 [file CTM2-11-e580-s008.TIF]

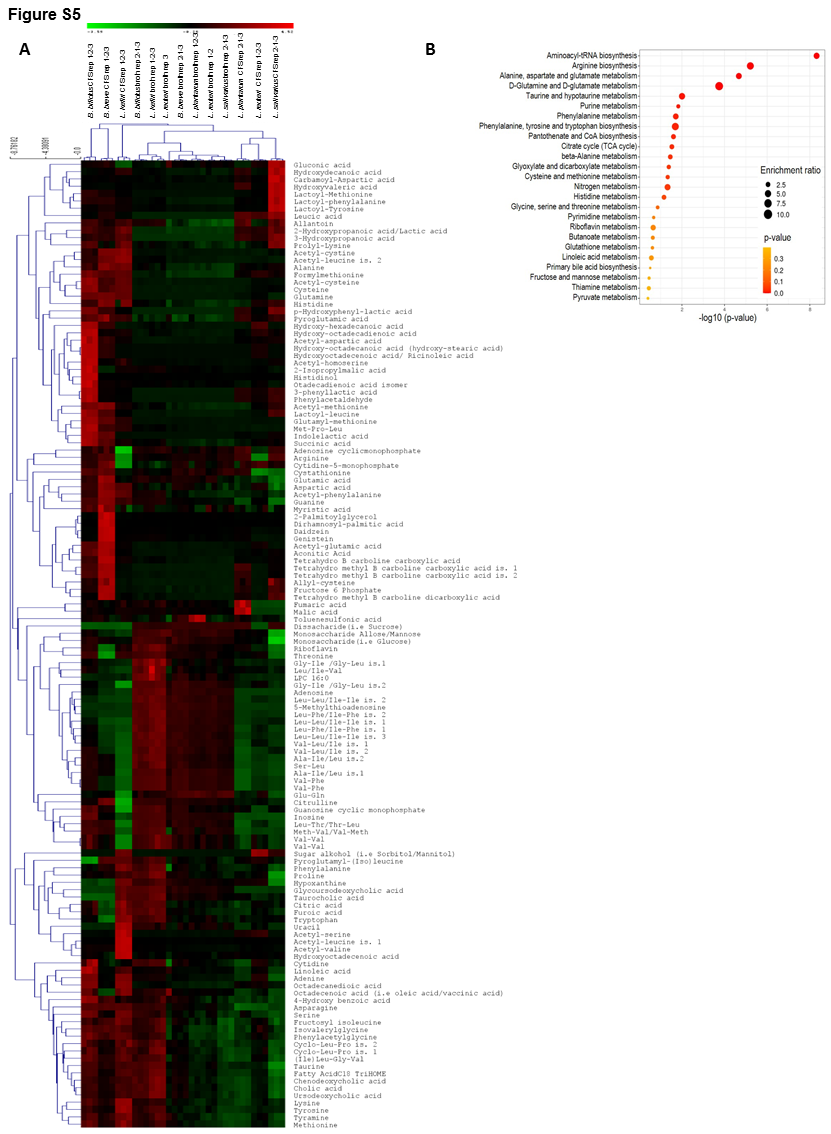

Supplement: Supplementary file 5 — Figure S5 [file CTM2-11-e580-s003.TIF]

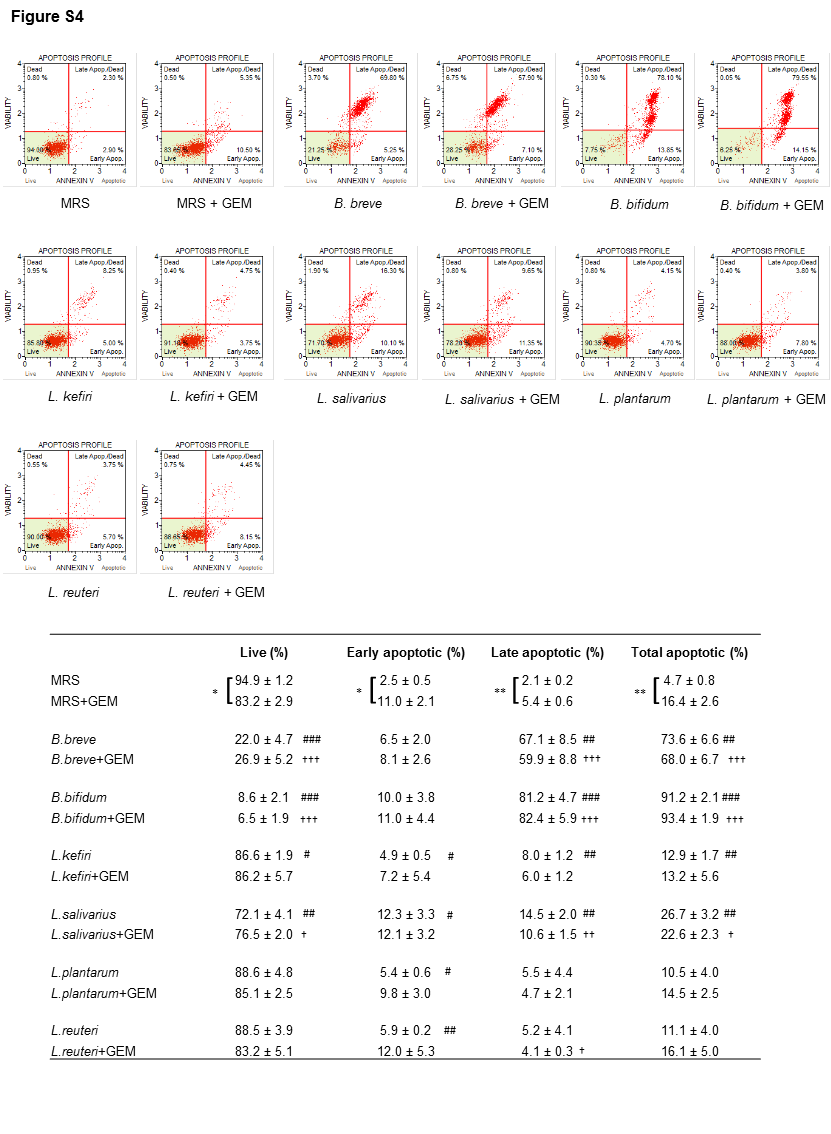

Supplement: Supplementary file 6 — Figure S4 [file CTM2-11-e580-s006.TIF]

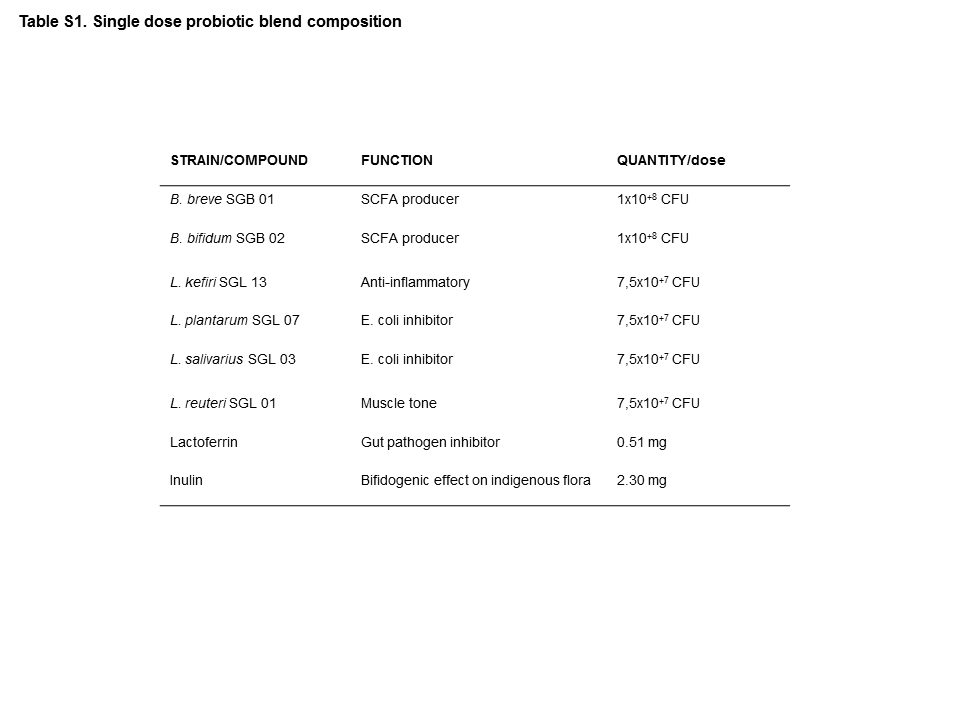

Supplement: Supplementary file 7 — Table S1: Single dose probiotic blend composition [file CTM2-11-e580-s009.TIF]

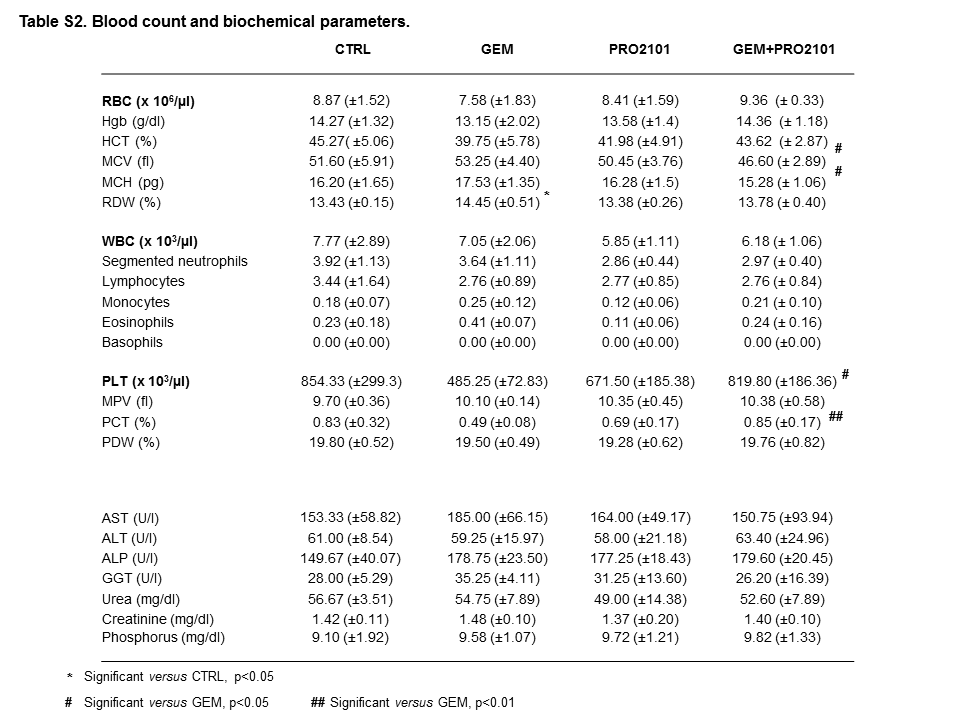

Supplement: Supplementary file 8 — Table S2: Blood count and biochemical parameters [file CTM2-11-e580-s001.TIF]
